# Valorization of Keratin Waste as a Functional Precursor for PLA/SBS Composite Adsorbent Films

**DOI:** 10.3390/polym18121483

**Published:** 2026-06-12

**Authors:** Maria Râpă, Raluca Nicoleta Darie-Niță, Andra Mihaela Predescu, Augusta Raluca Gabor, Cristian-Andi Nicolae, Carmen Gaidău, Corina Violeta Chiriță, Ramona Eugenia Popescu, Laurențiu Dincă

**Affiliations:** 1 Faculty of Materials Science and Engineering, National University of Science and Technology Politehnica Bucharest, 060042 Bucharest, Romania; maria.rapa@upb.ro (M.R.); andra.predescu@upb.ro (A.M.P.); 2 Physical Chemistry of Polymers Department, Petru Poni Institute of Macromolecular Chemistry, 41A Grigore Ghica Voda Alley, 700487 Iasi, Romania; 3 National Institute for Research & Development in Chemistry and Petrochemistry-ICECHIM, 202 Splaiul Independentei, 060021 Bucharest, Romania; raluca.gabor@icechim.ro (A.R.G.); cristian.nicolae@icechim.ro (C.-A.N.); 4 The National Research & Development Institute for Textiles and Leather, 16 Lucretiu Patrascanu Street, 030508 Bucharest, Romania; carmen.gaidau@incdtp.ro (C.G.); laurentiu.dinca@incdtp.ro (L.D.); 5 Biotechnical Systems Engineering Doctoral School, National University of Science and Technology Politehnica Bucharest, 313 Splaiul Independentei, 060042 Bucharest, Romania; corina.chirita1806@stud.isb.upb.ro

**Keywords:** keratin, wool waste, composite, adsorption, mechanical properties

## Abstract

This study investigated the valorization of keratin extracted from sheep wool waste for the preparation of PLA/SBS/Keratin composites as potential adsorbents for the removal of chromium (Cr) from synthetic water. A flexible formulation containing 75 wt% PLA and 25 wt% SBS was selected for the incorporation of 10 wt%, 20 wt%, and 30 wt% keratin. The morphology and structural characteristics of keratin and PLA-based composites were analyzed using SEM and FT-IR spectroscopy. The mechanical and thermal properties of the prepared composites were investigated using TGA and DMA analyses. The adsorption experiments revealed that keratin exhibited an adsorption capacity of 57.57 mg g^−1^ of Cr(VI) removal efficiency, while the PLA/SBS formulation containing 10 wt% keratin achieved a removal efficiency of total Cr of 55.41%. After three regeneration cycles, the removal efficiency decreased by approximately half of the total Cr removal.

## 1. Introduction

The rapid rise of industrialization has led to the generation of large volumes of untreated wastewater, causing irreversible environmental damage and posing significant risks to human health. Industries such as mining, manufacturing, and processing often produce effluents containing high concentrations of heavy metals that exceed permissible discharge limits, thereby requiring proper treatment before release into the environment [1,2]. Among these, chromium in its hexavalent form (Cr(VI)) is considered one of the most hazardous pollutants due to its high toxicity, carcinogenicity, teratogenic effects, bioaccumulation, and mobility in aquatic environments [3,4,5].

Cr(VI) is commonly detected in industrial effluents from electroplating and mining operations [6,7,8,9,10]. Due to its high solubility in water, Cr(VI) can easily migrate into wastewater systems and subsequently contaminate soil and groundwater [11,12,13,14,15,16,17]. Cr(VI) is classified as a human carcinogen by the United States Environmental Protection Agency and prolonged exposure can result in severe health conditions, including cancer, kidney damage, and even death [18]. Human exposure to chromium concentrations exceeding the safe limit of 0.2 mg L^−1^ may cause gastrointestinal symptoms such as nausea and diarrhea [16]. To protect public health, the World Health Organization (WHO) has established a guideline value of 0.05 mg L^−1^ for total Cr in drinking water.

The potential oxidation of Cr(III) to the more toxic Cr(VI) under extreme environmental conditions is the most significant concern [19]. For example, in the EU Member States, the Cr(VI) concentration emission from the tanning industry in water treatment plants ranges from 0.05 mg L^−1^ to 0.5 mg L^−1^ and from 0.1 mg L^−1^ to 3 mg L^−1^ for total Cr [20]. Therefore, it is essential to effectively remove chromium from industrial wastewater and ensure that its concentration is reduced below permissible limits before environmental discharge.

Among the established methods for removing heavy metal ions from aqueous solutions, chemical precipitation produces large amount of sludge and could lead to secondary pollution, while electrochemical reduction is associated with high operational costs and limited efficiency [21,22,23]. In contrast, membrane separation and adsorption are considered promising approaches due to their relatively simple operation, low cost, and high selectivity, and the potential for adsorbent reuse [24].

In recent years, various green adsorbents have been developed for the removal of Cr(VI) from wastewater, including straw biomass [25], coffee husk fiber [11], chitosan [26,27], fruit peel [28], porous carbon materials [29], cellulose [13], and clay [30]. In addition to adsorption, synergistic adsorption–photocatalytic processes such as CuS/TiO_2_ architectures have demonstrated high Cr(VI) removal efficiency [31]. Composite films have also shown great potential as efficient Cr(VI) adsorbents, representing a promising approach for the treatment of contaminated water. Some composites include bentonite and corn waste [8], polyaniline (PANi)-biochar [32], apricot kernel/polyaniline [12], chitosan (CS)/MnO_2_-perlite [33], cellulose/polylactic acid (PLA) blend [34], or polyacrylonitrile/polyethyleneimine/polypyrrole (PAN/PEI/PPy) composite nanofiber membrane [35]. Also, imprinted polymers could be used as synthetic adsorbents for heavy metals [36]. Ion-imprinted adsorbent systems designed for Cr(VI) feature special binding sites that match the size, shape, and charge of the target ions [37]. When combined with PLA, typically through methods such as electrospinning or bio-composite fabrication, they create environmentally friendly and mechanically strong membranes that can selectively remove Cr(VI) from complicated industrial wastewater, even in the presence of other ions [38].

PLA is a biodegradable polymer derived from the fermentation of starch and the condensation of lactic acid. It is made from 100% renewable resources such as corn, sugar beet, wheat, and other starchy products, making it a highly versatile polymeric matrix for various engineering applications [39,40,41,42,43]. However, PLA exhibits high rigidity, strength, and low ductility, which limits its practical use. To remove these disadvantages, PLA is commonly blended with environmentally friendly plasticizers such as polyethylene glycol (PEG) [44] or with other polymers, including polyhydroxybutyrate (PHB) [45] or styrene-butadiene-styrene (SBS) [46], or styrene-isoprene-styrene triblock copolymer [47]. Since standard PLA is inherently stiff and fragile, blending it with SBS greatly enhances its impact resistance, flexibility, and shape-memory capabilities [48].

Keratin, a protein-based biomass, has recently attracted attention as a potential material for the purification of water contaminated with heavy metals. It is abundant in sheep wool, chicken feathers, hair, and horns, with global production exceeding twelve million tons in 2020 [49]. Keratin possesses numerous thiol (–SH), carboxyl (–COOH), and amine (–NH_2_) groups, which confer hydrophilicity and excellent adsorption capacity. Currently, much of this biomass is discarded as combustion for fuel, but it is inefficient and polluting due to its high sulfur content (3–4 wt%). While keratin is well established in medical applications [50,51], it also has the ability to absorb and remove toxic substances, including heavy metal ions [52,53,54,55]. However, keratin has some limitations, including fragility and poor mechanical properties, which restrict its processing and applications. To overcome these limitations, keratin has been combined with other materials: Ag and TiO_2_ nanosols for photocatalytic degradation of rhodamine B dye [56], and nylon woven to prepare nanofiber membranes by electrospinning for filtration [57]. Similarly, mixtures of keratin with other synthetic polymers have been used to produce new materials with improved mechanical properties [58,59].

In this context, the innovation of the present study addresses the dual challenge of bio-waste management and water remediation by developing a novel flexible ternary composite film based on PLA, SBS, and keratin waste extracted from sheep wool. SBS was used for enhancing the elastic properties of the thermoplastic polymer. Despite these advances, there are limited studies investigating the use of keratin extracted from sheep wool as a modifier for PLA/SBS blends for Cr removal from aqueous solutions. The investigation integrates the Cr(VI) adsorption kinetics of the raw keratin with the comprehensive structural (FTIR), morphological (SEM), thermal (TGA), and thermo-mechanical (DMA) characterization of the polymeric matrices. This multidisciplinary approach enabled the selection of the optimal PLA/SBS formulation for efficient keratin incorporation, yielding eco-friendly filtration membranes designed for advanced toxic total chromium sequestration and complete pollutant retention. Ultimately, merging material engineering with active adsorption performance provides a vital blueprint that enhances the practical relevance and circular economy potential of functional biocomposites.

## 2. Materials and Methods

### 2.1. Materials

A commercial PLA 4032D grade commonly used for films and general-purpose applications (NatureWorks LLC, Minnetonka, MN, USA) was used as the polymeric matrix due to its biodegradability, good film-forming ability, and mechanical properties.

The starred SBS block-copolymer synthesized by anionic polymerization, according to our previous paper [60], was used as an elastomeric modifier to enhance the flexibility of films. It was characterized by a polystyrene (PS) content of 31.8 wt% and a molecular weight of 190,000 g mol^−1^.

Keratin extract (K), as powder, was kindly provided by the Leather Research Department (The National R&D Institute for Textiles and Leather, Bucharest, Romania) and was incorporated into PLA/SBS formulation as an active functional component for capturing and reducing the total Cr. Keratin with a molecular weight of 13 kDa was extracted from sheep wool waste, contributing to the circular economy approach, according to the previous reported methods [61]. Briefly, the wool waste was degreased in alkaline solution at 40 °C for 2 h, soaked, squeezed, chopped, and hydrolyzed with sodium hydroxide at 80 °C for 4 h.

Chloroform, purchased from Chimreactiv SRL, Bucharest, Romania (minimum purity 99%, density 1.485 g cm^−3^), was used as the solvent. K_2_Cr_2_O_7_, H_2_SO_4_, H_3_PO_4_, and diphenylcarbazide (DPC) were of analytical grade. A 1000 mg L^−1^ total chromium standard solution was purchased from Merck KGaA (Darmstadt, Germany).

### 2.2. Preparation of the PLA/SBS/K Composites

The PLA, elastomer, and keratin polymeric systems obtained at this stage by the Petri dish casting process are presented in Table 1. The PLA/SBA 75/25 ratio was kept when adding the variable keratin amounts.

A 10% PLA solution was prepared by dissolving PLA pellets in chloroform using a magnetic stirrer at 600 rpm at a temperature of 55 °C. Similarly, a 10% elastomer solution was obtained by dissolving SBS in chloroform. By combining the two solutions according to Table 1, transparent and homogeneous PLA/SBS films were obtained, with flexibility increasing as the elastomer content increased. Keratin was incorporated at concentrations of 10%, 20%, and 30% only in the 75% PLA/25% SBS formulation. The purpose of including keratin in PLA/SBS films was to prevent the dissolving of pure keratin into aqueous media and to allow the easy regeneration of adsorbent.

### 2.3. Characterization

#### 2.3.1. Active Functional Component Investigation

Keratin extract was characterized by means of dry substance content, ash, protein content, and pH value. Also, size dimension, polydispersity index (PDI), zeta potential, and isoelectrical point values were estimated using a Zetasizer NanoZSP tool (Malvern Panalytical Ltd., Malvern, UK).

#### 2.3.2. Morphological Analysis by Scanning Electron Microscopy (SEM)

The morphology of the initial PLA/SBS and PLA/SBS/Keratin composites was examined with a Quanta 450 FEG SEM (FEI, Eindhoven, The Netherlands). SEM images were obtained at an acceleration voltage of 20–30 kV and 133 Pa using the ETD detector in high vacuum mode. The morphology and Energy Dispersive of X-rays spectroscopy (EDX) analyses for the regeneration study were carried out with a FEI Quanta 200 SEM (Eindhoven, The Netherlands).

#### 2.3.3. Fourier Transform Infrared Spectroscopy (FT-IR)

The spectral characteristics of the keratin extract and prepared films were analyzed using ATR-FT-IR with an INTERSPEC 200-X Spectrophotometer (Interspectrum, Tartumaa, Estonia). Three measurements were carried out over the spectral region of 3500–700 cm^−1^, at a resolution of 4 cm^−1^ using one bounce ZeSe ATR accessory with a pressure applicator.

#### 2.3.4. Thermogravimetric Analysis (TGA)

TGA analysis of PLA/SBS and PLA/SBS/K samples was performed with a TGA–Q 5000 IR instrument (TA Instruments, New Castle, DE, USA). Samples of 5–10 mg were introduced into a platinum 100 µL sample pan. Nitrogen (99.999%) 50 mL min^−1^ and synthetic air (99.999%) 50 L min^−1^ were used as purge gas 1 and purge gas 2, respectively. The samples were heated from room temperature (RT) to 700 °C with a ramp of 10 °C min^−1^; then gas 2 was selected and maintained isothermally for 5 min. The instrument software version was V.3.13, Build 261.

#### 2.3.5. Mechanical Tests (DMA)

Dynamic Mechanical Analysis (DMA) of PLA/SBS-based keratin samples was carried out using a Q800 DMA instrument (TA Instruments, New Castle, DE, USA) under multi-frequency strain conditions. Rectangular specimens (12.5 mm × 7.0 mm × 0.5 mm) were subjected to testing from −100 °C to 150 °C in film tension mode at 1 Hz. The applied parameters included a heating rate of 3 °C min^−1^, an oscillation amplitude of 15 μm, and a static force of 0.01 N. The evolution of storage modulus (E′) and loss factor (tan δ) with temperature was systematic analyzed. The glass transition temperature (T_g_) was defined as the temperature corresponding to the maximum tan δ. The instrument software version was V20.24, Build 43.

#### 2.3.6. Sorption Experiments of Cr(VI) and Total Cr

The adsorption of Cr(VI) ions from synthetic waters was analyzed via UV–Vis spectroscopy using an Orion UV–Vis AQUAMATE 8000 (Thermo Fisher Scientific Water Analysis Instrumentation, Chelmsford, MA, USA) spectrometer in the presence of a concentration of 2 mg mL^−1^ 1,5-diphenylcarbazide (DPC) in acetone as indicator, at pH 2 (acidified with H_3_PO_4_ and H_2_SO_4_). The reaction produced a characteristic Cr(DPC)_2_ red-violet complex in the presence of H^+^ ions.

**Figure 1:**
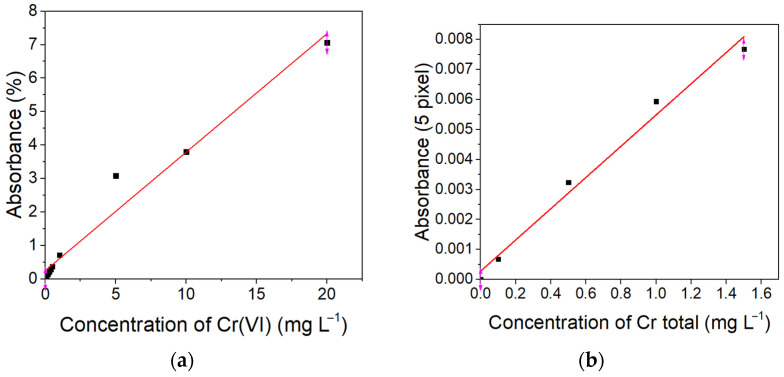
Calibration curve for Cr(VI) (0−20 mg L^−1^ at λ = 540 nm (**a**) and total Cr (0–1.5 mg L^−1^) at λ = 357.86 nm (**b**).

A stock solution containing 1000 mg L^−1^ Cr(VI) was prepared by dissolving 2.828 g K_2_Cr_2_O_7_ in distilled water. The calibration curve was obtained by measuring the absorbance at λ_max_ = 540 nm for working standards with concentrations of 0.1, 0.2, 0.3, 0.4, 0.5, 1, 5, 10, and 20 mg L^−1^, respectively (Figure 1a, y = 0.35403x + 0.2486, R^2^ = 0.9720). The 5 mg L^−1^, 10 mg L^−1^, and 20 mg L^−1^ working standards were diluted to ensure that the recorded absorbance values did not exceed 0.790.

Atomic absorption spectrometry (ContrAA 800, Analytik Jena AG, Jena, Germany) using the flame technique was used for the quantitative determination of total Cr removal by PLA/SBS/K composites. Calibrated total chromium standard solutions with concentrations of 0.1, 0.5, 1.0, and 1.5 mg L^−1^ were prepared from the stock solution. The resulting calibration curve is shown in Figure 1b (y = 0.00032 + 0.0051x, R^2^ = 0.9746).

The removal efficiency of Cr(VI) and total Cr were determined according to Equation (1).

(1) $$\mathrm{Cr}(\mathrm{VI})\;\mathrm{Removal}\;\mathrm{Efficiency}\;(\%)=\left(\frac{C_{i}-C_{e}}{C_{i}}\right)\times 100$$

where C_i_ and C_e_ are the initial and equilibrium concentrations (mg L^−1^) of the analyte under consideration (Cr(VI) or total Cr), respectively, in the sample; C_e_ is measured at liquid-phase equilibrium.

The amount of solute adsorbed from solution (q_e_) was calculated using Equation (2).

(2) $$q_{e}\;(\mathrm{mg}\;g^{-1})=\frac{\left(C_{i}-C_{e}\right)}{w}\times V$$

where V represents the volume of adsorbate (L), and w is the adsorbent mass (g).

For total Cr analysis, the PLA/SBS/Keratin samples were prepared as follows: a 166.9 mg sample was introduced into a Teflon microwave digestion vessel, together with a 30 mL solution of 1 mg L^−1^ total Cr, 3 mL of 65% HNO_3_, and 2 mL of 30% H_2_O_2_. The samples were digested using a scientific microwave (BERGHOF Microwave Digestion System, Berghof Products + Instruments GmbH, Eningen, Germany), according to the following temperature (°C)/power (%)/time (min) program: 160/80/5, 200/80/5, and 100/80/10. After completion of the digestion program, the digested sample was transferred into a 50 mL volumetric flask and diluted to the mark with distilled water. A blank digestion was performed under the same conditions as for the PLA/SBS/Keratin samples.

#### 2.3.7. Adsorption Kinetics

The adsorption kinetic parameters were calculated in the case of keratin adsorption using the pseudo-first-order Equation (3) and pseudo-second-order Equation (4).

(3) $$Log\left(q_{e}-q_{t}\right)=Log\left(q_{max}\right)-k_{1}t/2.303$$

(4) $$\frac{t}{q_{t}}=\frac{1}{k_{2}q_{max}^{2}}+\frac{t}{q_{max}}$$

where q_e_ and q_t_ are the adsorption capacities at equilibrium and time t (mg g^−1^), and k_1_ and k_2_ are the rate constants of the pseudo-first-order (min^−1^) and pseudo-second-order adsorption models (g mg^−1^ min^−1^).

Plots of Log(q_e_ − q_t_) versus contact time (t) and t/q_t_ versus contact time (t) calculated according to Equations (3) and (4) allow the evaluation of q_max_ and rate constants from the intercept and slope.

#### 2.3.8. Adsorption Isotherm Models

The Langmuir and Freundlich isotherm models were applied to evaluate the possible attractive forces between the Cr(VI) molecules in solution and the solid surface of keratin, according to Equations (5) and (6) [62].

C_e_/q_e_ = 1/(Q_max_ b) + C_e_/Q_max_ (5)

(6) $$\ln\left(q_{e}\right)=ln\left(K_{F}\right)+\frac{1}{n}ln\left(C_{e}\right)$$

where C_e_ is the concentration of the metal at equilibrium, mg L^−1^; q_e_ is the amount of Cr(VI) adsorbed per gram of the adsorbent at equilibrium, mg g^−1^; Q_max_ is the maximum adsorption quantity, mg g^−1^; b is the Langmuir constant, L mg^−1^, and n and K_F_ are constants related to Freundlich’s capacity and adsorption intensity, respectively.

Langmuir’s theory assumes that adsorbed molecules form a monomolecular layer on the surface of the adsorbent, and that once the adsorbed molecules have occupied a site, no further adsorption occurs and the interaction forces between the adsorbent and adsorbed molecules are negligible.

According to Freundlich’s theory, adsorption occurs heterogeneously (the adsorption sites on the adsorbent’s surface differ both in nature and in terms of energy). The Freundlich constants are determined from the slope and the y-intercept of the line by plotting ln(q_e_) against ln(C_e_).

## 3. Results and Discussions

### 3.1. Keratin Characterization

The main characteristics of keratin extract were dry substance of 96 ± 0.35%, ash content of 15 ± 0.24%, protein content of 70 ± 0.92%, and pH value of 7.00 ± 0.01.

**Figure 2:**
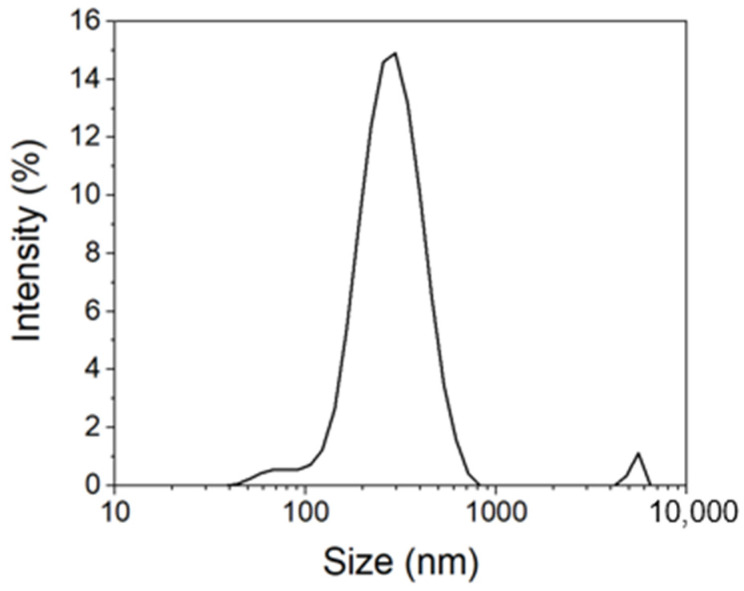
Size distribution of keratin dissolved in distilled water.

According to Zetasizer analysis, keratin indicates a complex and polydisperse system. It shows one intense peak at 296.7 ± 109.7 nm and two smaller peaks at 71.93 ± 14.02 nm and 5388 ± 317.3 nm (Figure 2). Zeta-average particle size is 280 nm, the PDI is 0.358, and the zeta potential is −29.0 ± 6.27 mV.

Its isoelectric point (pH_PZC_) is 3.81 (determined by the Zetasizer titration method), indicating that, at pH value of 3.81, the keratin surface is positively charged, making it suitable for electrostatic attraction with negatively charged species such as HCrO_4_^−^ and CrO_4_^2−^.

The adsorption experiments were strictly conducted at a controlled pH of 2.0, as this acidic threshold represents the optimum thermodynamic and electrostatic window for Cr(VI) removal using keratin [63]. The strong pH-dependence of the process is governed by sorbate speciation and adsorbent surface charge. Thus, in highly acidic aqueous environments (pH 1.0−3.0), Cr(VI) predominantly exists in the form of hydrochromate anions (HCrO_4_^−^). Compared to the chromate (CrO_4_^2−^) and dichromate (Cr_2_O_7_^2−^) forms that prevail at higher pH values, the HCrO_4_^−^ anion possesses a lower adsorption free energy, making it much easier to be chemically bound to active sites [64]. Also, at low isoelectrical points (pH < 3.81), the high concentration of H_3_O^+^ induces intense protonation of the amino (−NH_3_^+^) and other basic functional groups distributed along the polypeptide chains of the keratin matrix. This massive protonation generates a dense, highly positive surface net charge. Consequently, strong electrostatic attraction is established between the positively charged keratin surface and the negatively charged HCrO_4_^−^ anions, maximizing the adsorption capacity (q_e_). At higher pH values, the deprotonation of amino groups and the competitive repulsion from hydroxyl ions (OH^−^) severely suppress the adsorption efficiency, justifying why pH 2.0 was selected as the exclusive operating condition for this study.

### 3.2. SEM Analysis

**Figure 3:**
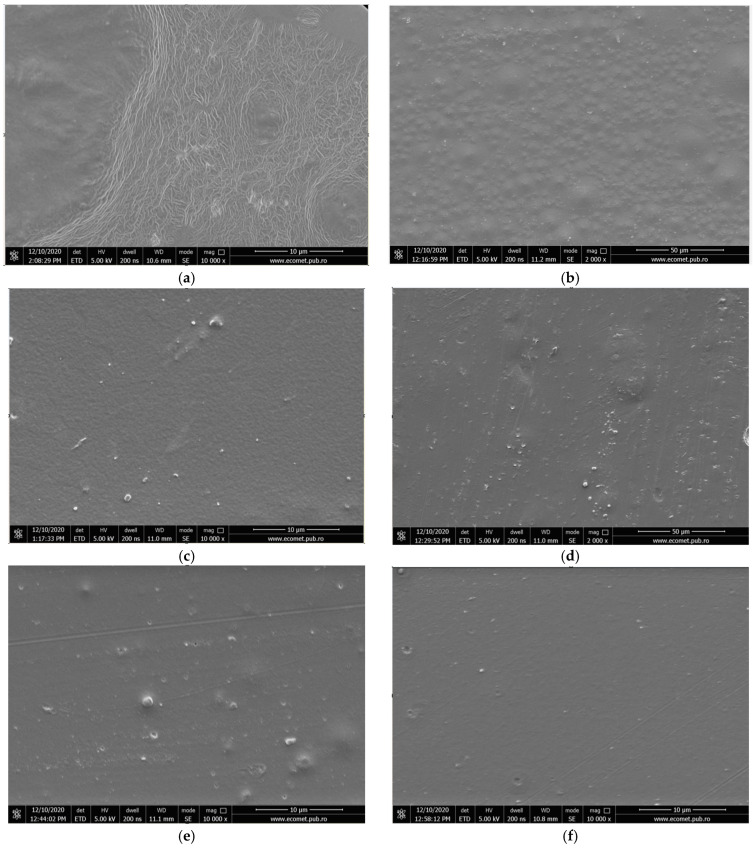

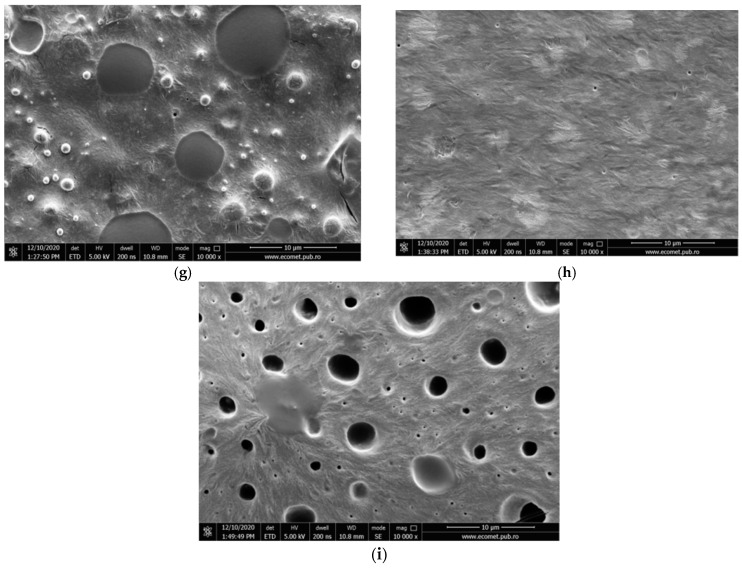
SEM images for keratin (**a**), PLA film (**b**), SBS film (**c**), PLA/SBS 25/75 film (**d**), PLA/SBS 50/50 film (**e**), PLA/SBS 75/25 film (**f**), PLA/SBS/K 10 film (**g**), PLA/SBS/K 20 film (**h**), and PLA/SBS/K 30 film (**i**).

Figure 3 shows SEM images of keratin and PLA/SBS polymer systems containing varying amounts of keratin.

At a magnification of 10,000×, keratin reveals a network of fibrous structures, with irregular and rough surfaces typical for keratin protein assemblies (Figure 3a). This morphology suggests a high surface area with abundant active sites, which can be advantageous for adsorption applications.

Figure 3b–f illustrate a smooth, dense, and uniform surface of PLA, SBS, and PLA/SBS blends, respectively, with minimal porosity and few surface irregularities.

Figure 3g,i show a granular, porous surface morphology of a PLA/SBS blend with 10 wt% and 30 wt% keratin incorporated as dispersed particles. The distribution appears fairly uniform but indicates some degree of phase incompatibility. These micro-voids represent spaces previously occupied by keratin and may be formed due to the solvent evaporation [65]. The rough, cratered surface and micro-voids increase the specific surface area, providing more active sites for pollutants to attach. This generally enhances the adsorption capacity for heavy metal pollutants. A similar morphology was reported for keratin extracted from the chicken feathers [66]. Incorporation of 20 wt% keratin into PLA/SBS 75/25 results in a good dispersion and stronger interfacial adhesion between components (Figure 3h). Keratin is better embedded within the polymeric phases due to its hydrophilicity [59].

**Figure 4:**
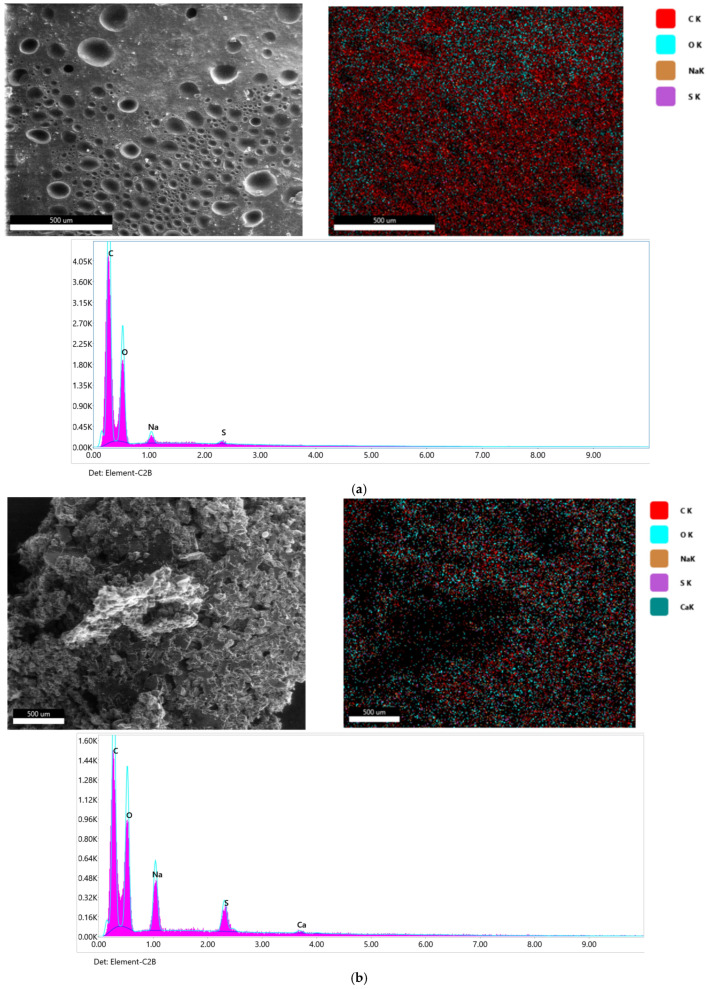
EDX spectroscopy of initial samples. PLA/SBS/K 20 (**a**) and PLA/SBS/K 30 composites (**b**) (magnification 100 ×).

Elemental composition analysis carried out with EDX spectroscopy for PLA/SBS/K 20 and PLA/SBS/K 30 samples is illustrated in Figure 4a,b, and Table 2.

The composites containing keratin show the presence of C, O (elements specific to organic materials), S (a characteristic element of keratin proteins), and Na and Ca (mineral constituents naturally associated with keratin). A similar elemental composition (C, O, S, and Na) was reported for keratin extracted from chicken feathers by Manju and Sharma [67]. Increasing the keratin content from 20 wt% to 30 wt% resulted in an increase in S content from 0.4 wt% to 3.0 wt%. The enhanced S signal is attributed to the cysteine-rich structure of keratin, which contains disulfide crosslinks characteristic of keratinous materials. The higher S concentration in PLA/SBS/K 30 provides strong evidence for the successful incorporation of a greater amount of keratin into the composite.

### 3.3. ATR-FT-IR Analysis

**Figure 5:**
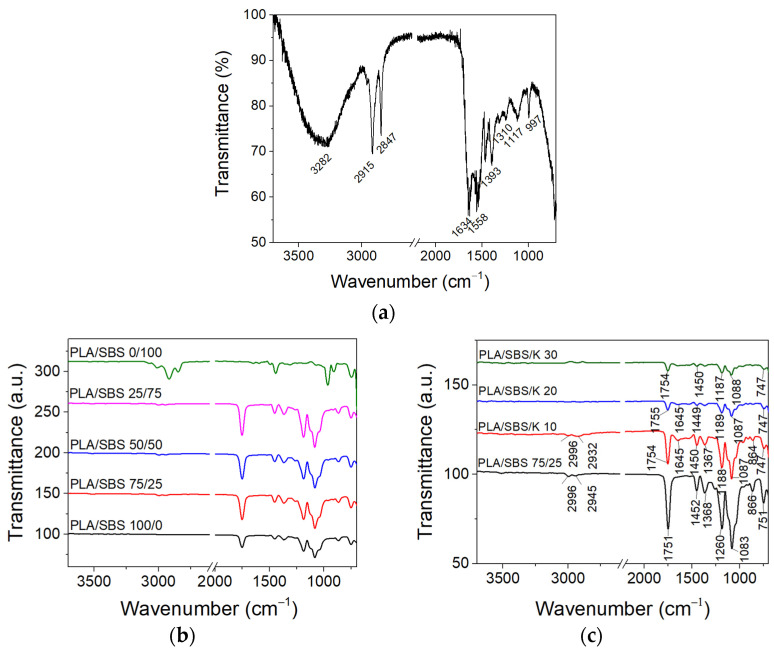
ATR-FT-IR spectra for keratin (**a**), PLA film, SBS film, PLA/SBS films, and PLA/SBS films (**b**), and PLA/SBS 75/25 containing different amounts of keratin (**c**).

Figure 5a–c show the ATR-FT-IR spectra of keratin, neat PLA, SBS, PLA/SBS blends, and PLA/SBS/K composites.

The FT-IR analysis of keratin reveals prominent bands at 3282 cm^−1^, 2915 cm^−1^, 2847 cm^−1^, 1634 cm^−1^, 1558 cm^−1^, 1393 cm^−1^, and 1310 cm^−1^ due to the NH-stretching (Amide A), symmetric stretching of the –CH_2_– group (Amide B), the α-helix structure of the keratin protein (Amide I band), the bending vibration of the –NH group coupled with C–N (Amide II), and the stretching vibration of the C–H group (Amide III). The band at 1117 cm^−1^ is attributed to the sulfur oxide functional groups as indicative of cysteine oxidation, while that at 997 cm^−1^ is associated with the cysteine acid group [68,69] (Figure 5a).

The characteristic PLA absorption bands are observed at 1748 cm^−1^ (C=O stretching vibration of the ester group), 1452 cm^−1^ and 1365 cm^−1^ (–CH_3_ asymmetric bending vibrations), and 1180 cm^−1^ (CH_3_ symmetric bending vibrations) [42] (Figure 5b).

For SBS, typical absorption bands appear at 753 cm^−1^, assigned to C=C stretching vibration of the styrene units; 910 cm^−1^ and 960 cm^−1^, corresponding to =C–H out-of-plane bending vibrations of the butadiene units; and 2848 cm^−1^ and 2915 cm^−1^, attributed to symmetric –CH_2_– stretching and asymmetric –CH_3_ stretching vibrations, respectively [60] (Figure 5b).

The characteristic absorption bands of both components in PLA/SBS blends are retained without the appearance of new bands, indicating that no chemical reactions occurred between PLA and SBS and that the system is mainly governed by physical blending. A shift of the carbonyl band to a high wavenumber is observed for the PLA/SBS 75/25 blend (1751 cm^−1^) compared with PLA/SBS 50/50 (1745 cm^−1^), while the C=C stretching vibration of the styrene groups of the SBS moved to a lower wavenumber (from 910 cm^−1^ to 865 cm^−1^) for PLA/SBS 50/50 and 967 cm^−1^ for PLA/SBS 25/75 blends (Figure 5b).

For PLA/SBS/K composites, the specific bands of components disappeared (a specific band of keratin at 3282 cm^−1^), overlapped, shifted (for example, Amide I of keratin), or decreased in the intensity with the introduction of keratin (for example, the characteristic vibrations of the SBS phase, mainly associated with the butadiene units), indicating hydrogen bonding between PLA and keratin, which are expected to enhance surface polarity and adsorption affinity toward Cr ions (Figure 5c).

### 3.4. TGA Analysis

**Figure 6:**
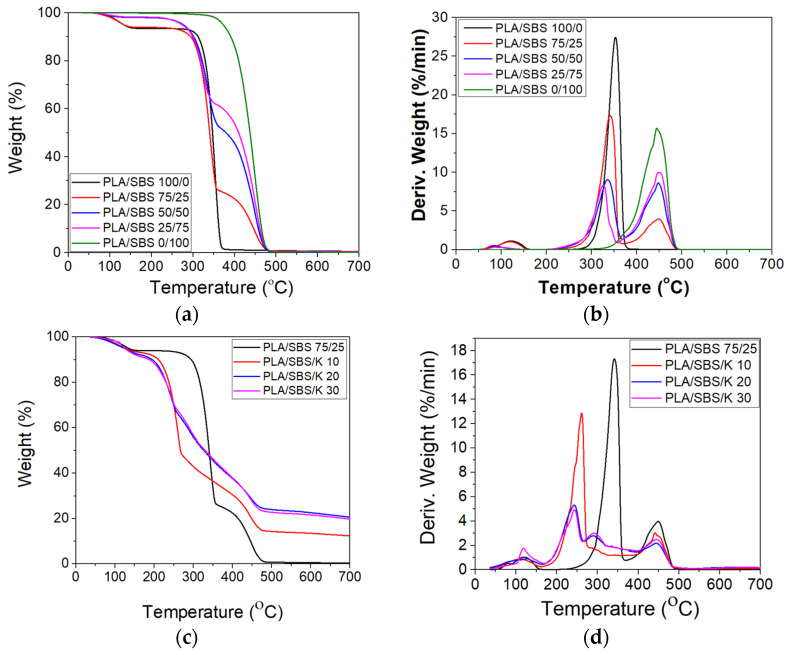
Thermogravimetric (TGA) thermograms. Weight loss (%) for neat PLA, SBS, PLA/SBS 75/25, PLA/SBS/50/50, PLA/SBS25/75 (**a**) and their derivative weights (%/min) (**b**). Weight loss (%) for PLA/SBS 75/25, PLA/SBS/K 10, PLA/SBS/K 20, and PLA/SBS/K 30 composites (**c**) and their derivative weights (%/min) (**d**).

Figure 6a,b illustrate the thermal stability of PLA, SBS, PLA/SBS, and PLA/SBS/K formulations.

The degradation temperatures and weight loss values are shown in Table 3.

The degradation behavior of PLA/SBS blends reveals a two-stage decomposition process, influenced progressively with increasing SBS content. Neat PLA (PLA/SBS 100/0) exhibits one major degradation step between 270 and 370 °C, with a maximum degradation temperature (T_max3_) of 353 °C and a weight loss of 91.33%, characteristic of PLA thermal decomposition [70]. These results show that blending PLA and SBS reduces thermal stability, likely because many unstable interfaces form and are vulnerable to thermal energy, as also demonstrated by Wang et al. [71]. As the SBS content increases, the main degradation peak shifts toward lower temperatures, from 353 °C for pure PLA to 326 °C for PLA/SBS 25/75, indicating a reduction in thermal stability. At the same time, the degradation occurring between 370 °C and 545 °C becomes more pronounced, corresponding to the thermal decomposition of the SBS phase. Pure SBS shows its main degradation at approximately 444 °C with a weight loss of 95.36%, confirming its higher thermal resistance compared with PLA (Table 3).

The incorporation of keratin substantially modifies the degradation pattern of the composites. PLA/SBS/K 10, PLA/SBS/K 20, and PLA/SBS/K 30 composites exhibit an additional degradation stage between 170 and 270 °C, with T_max2_ values around 243–261 °C. This degradation step is attributed to the decomposition of keratin components, including peptide chains and low-molecular-weight fractions [72,73]. The presence of this stage confirms the successful incorporation of keratin into the polymer matrix.

The keratin-containing composites also show a reduction in the weight loss associated with the PLA degradation region (270–370 °C) compared with the PLA/SBS blends without keratin. This behavior suggests that keratin partially alters the thermal decomposition pathway of the matrix, possibly through intermolecular interactions between keratin functional groups and the polymer chains. Thermal degradation of keratin clearly affects the overall properties, including the thermal degradation of the whole PLA/SBS composite containing it. Similar thermo-degradation findings were reported by Wei et al. [74] for keratin/gelatin/glycerin/curcumin film, where the second degradation step is observed in the temperature range of 130–400 °C, with a maximum degradation at 365 °C, due to the degradation of keratin chain backbone, while the third step of degradation in the 400 and 800 °C range is related to the intramolecular hydrogen bond in the keratin chain structure.

An important observation is the substantial increase in residual mass at 700 °C for the keratin-containing samples. While PLA/SBS blends leave negligible residues (<1%), the PLA/SBS/K composites show residues between approximately 12–21% in N_2_ and 8–15% in air. The higher char formation is associated with the proteinaceous structure of keratin, which promotes carbonaceous residue formation during thermal degradation. Among the composites, PLA/SBS/K 20 exhibits the highest residue values, suggesting enhanced char-forming ability and improved thermal resistance at elevated temperatures.

### 3.5. DMA Analysis

**Figure 7:**
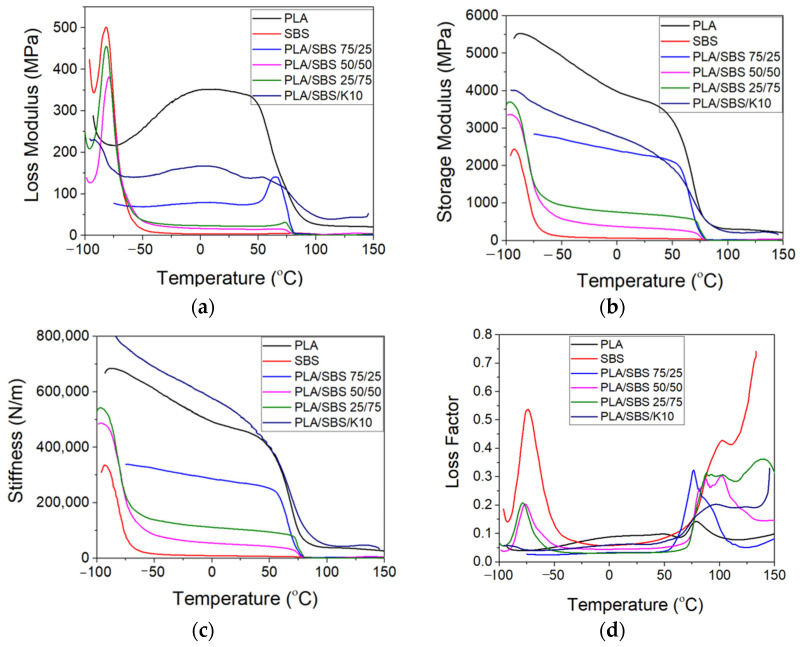
Dynamic mechanical analysis (DMA) results for the investigated samples. Loss modulus (E″) (**a**), Storage modulus (E′) (**b**), Stiffness (**c**), and Tan δ (**d**).

The effect of keratin 10 wt% and SBS 25 wt%, 50 wt%, and 75 wt% on the viscoelastic properties of PLA blends was investigated by DMA analysis, in the region of −100 °C to 150 °C; see Figure 7a–d.

Figure 7a shows the temperature dependence of the loss modulus (E″) for neat PLA, SBS, their blends, and the PLA/SBS/K10 composite, providing insight into the viscoelastic transitions and molecular mobility of the materials. Neat PLA exhibits a broad relaxation peak in the mid-temperature range (around 0–60 °C), corresponding to its glass transition region. The relatively high E″ indicates enhanced chain mobility and damping capacity during transition from glassy to rubbery states.

At a temperature of ~60 °C, the incorporation of SBS into PLA reduces the storage modulus and overall stiffness of the blends, reflecting the plasticizing and flexibility-enhancing effect of SBS (Figure 7b,c). PLA shows a sharp decrease in stiffness beyond 50–60 °C, while the E′ of SBS also declines with increasing temperature as it moves from a glassy to a rubbery state following the glass transition. SBS copolymers are characterized by a two-phase structure, displaying two distinct glass transition temperatures (T_g_), as also observed from Figure 6d, the first related to the polybutadiene block (PB) at approximately −80 °C and another one for the polystyrene block (PB) (~100 °C) [75]. In this study, pure SBS shows the lowest E′ (0.7899 MPa) and relatively high tan δ (0.7373), confirming its highly flexible and damping-dominant elastomeric nature. SBS shows a sharp and intense peak at lower temperatures (around −80 to −50 °C), which is associated with the glass transition of the butadiene soft phase [60]. Neat SBS exhibits the highest damping response (tan δ = 0.5416 at a temperature of −72.94 °C) and the highest loss modulus (506.3 MPa) at the temperature of −81.22 °C, indicating strong viscous energy dissipation but low structural rigidity; see Figure 7d.

In comparison with the PLA/SBS 75/25 blend, the addition of keratin results in an increase in both E′ and stiffness, respectively, indicating a reinforcing effect attributed to keratin–polymer interactions that restrict chain mobility and improve load-bearing capacity. As SBS content increases in the PLA/SBS blends, there is a clear reduction in both E″ and tan δ, reflecting a transition toward more elastic and less dissipative behavior. The PLA/SBS 50/50 and PLA/SBS 25/75 blends show intermediate damping characteristics, while the PLA/SBS 75/25 blend exhibits a pronounced drop in tan δ (0.208 at the temperature of −78.43 °C), indicating a more rigid, less viscoelastic response. The PLA/SBS 50/50 system shows two peaks in loss factor evaluation around 100 °C due to equal amount of PLA and SBS, displaying the T_g_ of the main components, slightly changed from ~70 °C for PLA and one for the PS block (PB) (~100 °C) in SBS, respectively. The PLA/SBS/K system displays a single large loss factor peak around 100 °C, showing improved compatibility of the PLA and SBS phases. This broad peak in tan δ is related to superior vibration or noise-damping characteristics, particularly observed in elastomers or specially designed composites [76].

The PLA/SBS/K 10 composite shows slightly increased E″ (230.8 MPa at the temperature of −92.15 °C) compared to the PLA/SBS 75/25 blend, along with a marginal change in tan δ. This suggests that keratin contributes to enhanced interfacial interactions, leading to restricted chain mobility while improving thermal stability, as reflected by the shifted transition temperature.

### 3.6. Adsorption Experiments

#### 3.6.1. Effect of Keratin Adsorbent Dose

**Figure 8:**
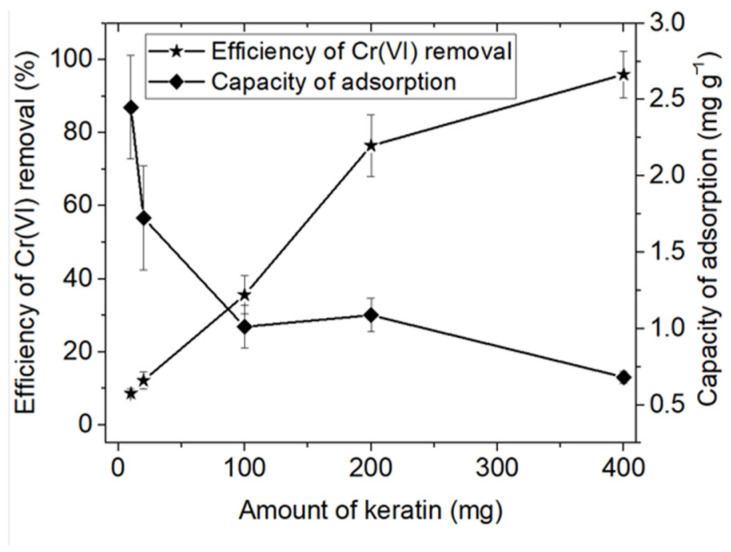
Efficiency of Cr(VI) removal and adsorption capacity (q_e_) by keratin through UV–Vis spectrometry. Experimental conditions: λ = 540 nm, concentration of the Cr(VI) solution of 20 mg L^−1^, pollutant volume of 10 mL, mass of adsorbent of 10 mg, 20 mg, 100 mg, 200 mg, and 400 mg, pH = 2.

The Cr(VI) retention efficiency and the adsorption capacity of keratin at different amounts (10–1000 mg) and contact times (30 min, 60 min, and 90 min) are illustrated in Figure 8.

Figure 8 shows that as the amount of adsorbent increased from 10 mg to 400 mg, the pollutant removal efficiency improved. A significant efficiency of Cr(VI) removal (95%) is recorded when 400 mg of adsorbent was used. Billahet al. [33] also reported a maximum adsorption capacity of Cr(VI) at a concentration of adsorbate of 1 g L^−1^ in the case of CS/MnO_2_-perlite composite. In contrast, the highest amount of metal retained per 1 g of adsorbent (2.45 mg g^−1^) was observed in the case of 10 mg keratin in contact with the 20 mg L^−1^ pollutant solution. This behavior is due to a higher number of active binding sites, such as the –NH_2_ and –SH groups of keratin. Conversely, at 400 mg of keratin, many sites remained unsaturated, and the adsorption capacity dropped significantly. The same trend was observed by Hossain et al. [9] when keratin film was used as an adsorbent for the removal of Cr(III)) from wastewater samples.

#### 3.6.2. Kinetic Adsorption

**Figure 9:**
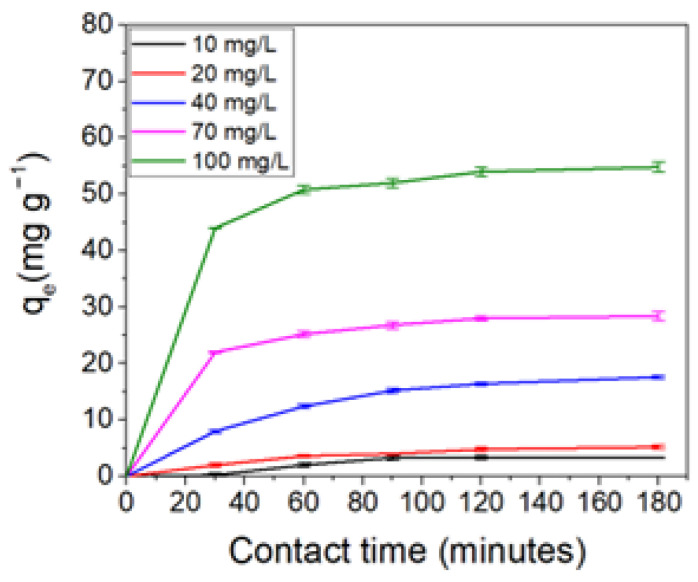
Cr(VI) retention efficiency of keratin using the UV–Vis technique. Experimental test conditions: λ = 540 nm, solute concentrations in the range of 0–100 mg L^−1^, solution volume of 40 mL, keratin mass of 10 mg, contact times up to 180 min, pH = 2.

Figure 9 shows the effect of pollutant concentration on the amount of Cr(VI) retained by one gram of keratin adsorbent.

A rapid increase in adsorption capacity (q_e_) is observed within the first 30 min, which can be attributed to the availability of abundant active sites on the adsorbent surface. The highest adsorption capacity (~50 mg g^−1^) by 10 mg of keratin was observed at a pollutant concentration of 100 mg L^−1^ at a contact time of 90 min (Figure 9). This suggests the presence of an optimum concentration at which the adsorbent operates most efficiently. Up to 80 min, the driving force for mass transfer is adequate without causing saturation of the active sites.

The kinetic parameters for Cr(VI) adsorption at different concentrations (10–100 mg L^−1^) are summarized in Table 4.

The pseudo-first-order model shows discrepancies between the calculated (q_max_) and experimental (q_e_) values, with correlation coefficients (R^2^) ranging between 0.808 and 0.999, which suggests that this model does not adequately describe the adsorption process at all concentrations studied. As shown in Table 4, the pseudo-second-order kinetic model provides the best fit for the adsorption of Cr(VI) in the presence of keratin in the concentration range from 10 mg L^−1^ to 100 mg L^−1^ (high value of the regression coefficient R^2^). Similar behavior has been reported for other adsorbents using coffee husk fiber composite (R^2^ = 0.9942) [11], chitosan-coated coconut shell composite (R^2^ = 0.999) [6], apricot kernel/polyaniline composite [12], and cellulose composite aerogel (R^2^ = 0.999) [13]. These findings suggest that chemosorption involving the formation of chemical bonds between the adsorbent and Cr(VI) is the rate-limiting step of the process. Other systems where the pseudo-first-order model provides a better fit, such as the bentonite and corn waste composite (R^2^ = 0.968) [8] and CS/MnO_2_-perlite composite [33], indicated a mechanism dominated by physical adsorption or diffusion processes that depended more on the solute concentration.

Table 5 illustrates a comparison of the adsorption capacity of different adsorbents reported in the literature for Cr(VI) retention.

Among the investigated materials presented in Table 5, the keratin extract exhibits a moderate adsorption capacity for Cr(VI) removal, comparable to that of plant-derived and polysaccharide-based materials.

#### 3.6.3. Adsorption Isotherms

**Figure 10:**
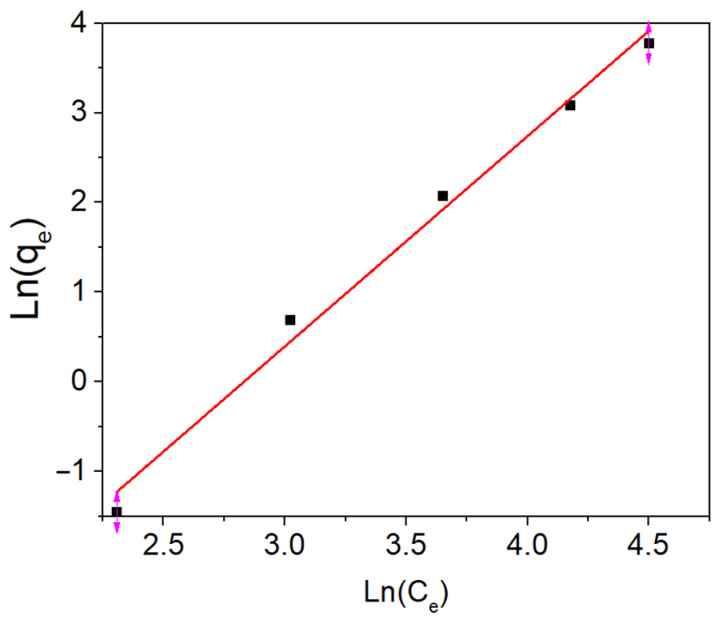
Experimental conditions: different pollutant concentrations (10–100 mg L^−^); adsorbent quantity: 10 mg; pollutant solution volume: 40 mL; contact time: 30 min, pH = 2.

The Freundlich adsorption isotherm is shown in Figure 10.

The Langmuir model is not suitable for keratin adsorption due to the negative slope. Based on regression analysis (Figure 10), it was found that the experimental data best fit the adsorption equilibrium described by the Freundlich isotherm (R^2^ = 0.987). This high correlation coefficient demonstrates that the adsorption occurs on a heterogeneous surface characterized by a non-uniform distribution of adsorption heat over the active sites, typical of complex biopolymer matrices. The calculated 1/n value is 2.3, indicating that adsorption involves strong interactions between keratin molecules. As the initial sites become occupied, the presence of already adsorbed keratin molecules modifies the surface properties, enhancing the affinity for subsequent molecules and facilitating multi-layer formation. Functional groups, such as amino (−NH_2_), carboxyl (−COOH), and thiol (−SH) groups, inherently to their polypeptide structure, not only bind Cr(VI) by electrostatic attraction and complexation, but also engage in strong intermolecular interactions (such as hydrogen bonds) among the protein chains.

Furthermore, the Freundlich capacity constant (K_F_) is 1.3 × 10^−3^ mg g^−1^ (L mg^−1^)^1/n^, reflecting the baseline adsorption capacity of the system under the investigated experimental conditions. Similar behaviors, where the adsorption equilibrium preferentially aligns with the Freundlich model, have been widely reported for various alternative materials used for chromium removal. These include bentonite and corn waste adsorbent [8], apricot kernel/polyaniline composites [12], and cellulose-based biocomposite blended with PLA [34]. Tejada-Tovar et al. [34] attributed this phenomenon to the prominent role of surface-bound functional groups, especially carboxyl and hydroxyl groups present on PLA and cellulose matrices. These groups establish highly active chemical sites on the biocomposite surface, driven by electrostatic interactions and coordination bonds with the oxyanions of Cr(VI).

#### 3.6.4. Removal of Total Cr in the Presence of PLA/SBS/K Composites

To prevent the dissolution of keratin in aqueous media, the strategy was to integrate it into a PLA/SBS composite (75/25 wt% ratio). The total Cr removal efficiency and regeneration tests were evaluated using atomic absorption spectrometry. After evaluating the total Cr removal efficiency, the samples were immersed in a 1 M NaOH solution for 2 h, followed by washing and drying. Subsequently, the regenerated samples were immersed in 30 mL of a 1 mg L^−1^ total Cr solution for 1 h, under magnetic stirring (250 rpm) at room temperature.

**Figure 11:**
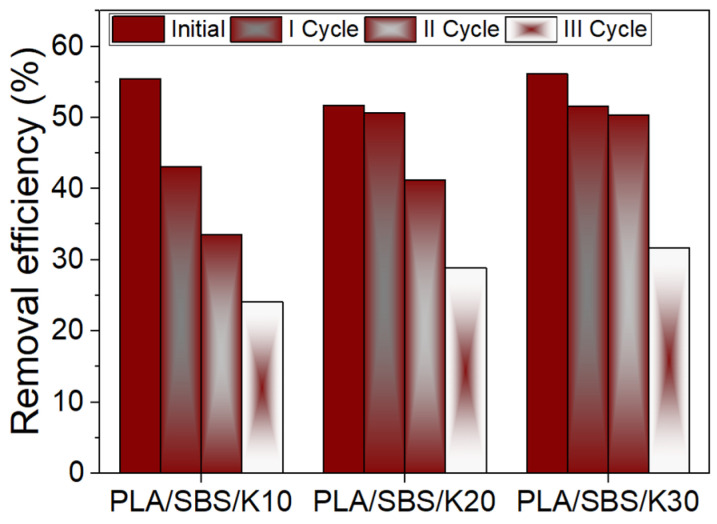
Total Cr removal efficiency and the regeneration tests for the PLA/SBS/K composites using atomic absorption spectrometry. Experimental test conditions: Solute concentration of 1 mg L^−1^, solution volume of 30 mL, keratin mass of 166.9 mg.

Figure 11 plots the total Cr removal efficiency and regeneration performance of PLA/SBS/K composites.

The PLA/SBS/K 10, PLA/SBS/K 20, and PLA/SBS/K 30 composites exhibit relatively high removal efficiencies, with values between 51% and 56%. The PLA/SBS/K 10 sample records a removal of 55.1% of total Cr, and is considered a potential material for the treatment of contaminated water samples. It has been reported that the Cr removal efficiency can be maintained due to the presence of hydrophobic groups on the keratin surface which prevent mixing in aqueous solutions [55]. However, in another study [78], extruded PLA films coated with PANi and PPy showed high Cr(VI) removal efficiencies at pH 2, with PLA-PANi achieving an efficiency of 97.39% and PLA-PPy reaching 92.15%.

The removal of Cr using PLA/SBS/K composites can occur through a combination of physical adsorption and chemisorption. Physical adsorption occurs by trapping Cr(VI) and Cr(III) in the porous network of PLA/SBS/K samples. The reduced Cr(III) ions are subsequently immobilized on the keratin matrix by complexation with amino and carboxyl groups, forming stable coordination bonds. This dual mechanism, adsorption coupled with reduction, enhances the overall removal efficiency and prevents the release of Cr into the environment. The adsorption properties of wool keratin are mainly attributed to the side-chain functional groups of its constituent amino acids, especially glutamic acid, aspartic acid, arginine, and lysine, which provide active sites for interaction with metal ions [57]. It is possible that the chemisorption occurs at the carboxyl groups of keratin and PLA ester.

**Figure 12:**
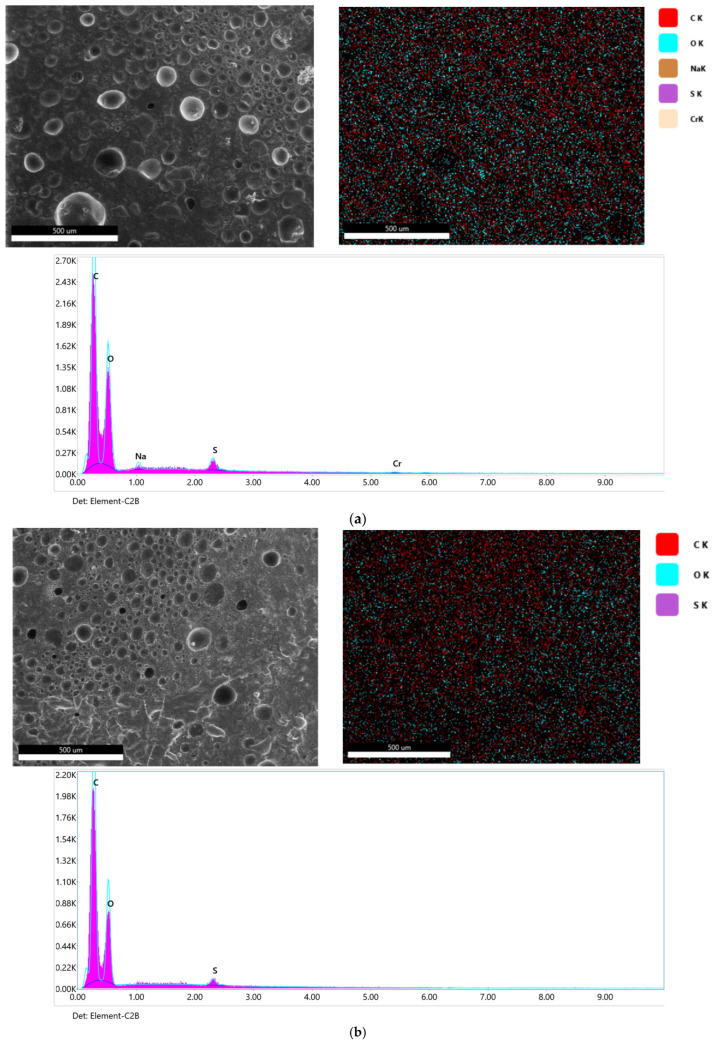
EDX spectroscopy of PLA/SBS/K 20 sample. Adsorbed Cr (**a**) and desorbed Cr (**b**).

**Figure 13:**
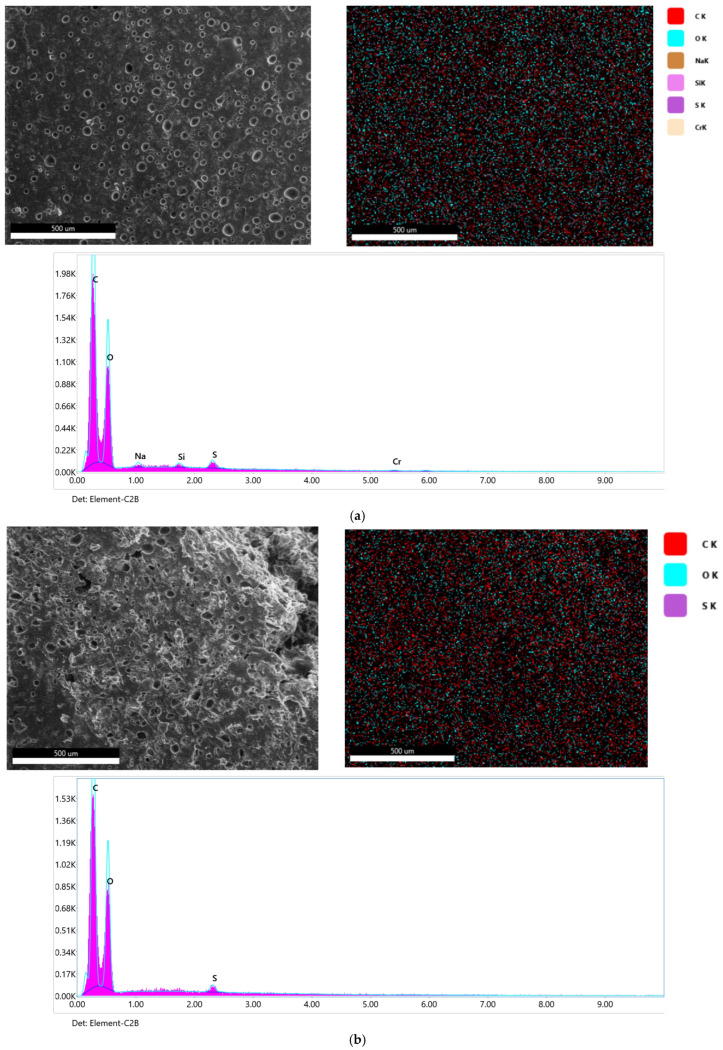
EDX spectroscopy of PLA/SBS/K 30 sample. Adsorbed Cr (**a**) and desorbed Cr (**b**).

Figure 12 and Figure 13 show the EDX profile of adsorption/desorption of Cr by PLA/SBS/K 20 and PLA/SBS/K 30 composites. Table 6 contains the elemental composition of these composites based on the EDX data provided.

EDX analysis revealed the presence of Cr on the surface of PLA/SBS/K 20 and PLA/SBS/K 30 composites after adsorption, with Cr contents of 0.4% and 0.3%, respectively. After the desorption process, Cr was no longer detected, indicating the successful regeneration of the adsorbents and the efficient removal of the adsorbed Cr species.

The increase in oxygen content from 36.5 wt% to 40.5 wt% during Cr adsorption indicates that oxygen-containing functional groups on the keratin-modified composite play a significant role in chromium binding. The oxygen-rich surface generated by the higher keratin loading provides additional active sites for interaction with Cr species. This observation, together with the detection of Cr on the adsorbent surface after adsorption and its disappearance after desorption, supports a mechanism involving the participation of oxygenated functional groups in the absorption of chromium. The subsequent decrease in sulfur content after desorption suggests partial dissolution or leaching of keratin, indicating that some keratin-derived functional groups may be lost during the regeneration process. After successive regeneration cycles, the removal efficiencies decrease noticeably, explaining the partial loss of active sites, from 43 to 51%, 33 to 41%, and 34 to 31% in the first, second, and third regeneration cycles, respectively. The reasons for the substantial decrease in the regeneration performance of the adsorbents could be the hydrophilicity of keratin, which leads to the loss or deactivation of adsorption sites (–NH_2_, –COOH, –OH, and disulfide groups), incomplete desorption of Cr, and possible leaching of keratin particles during regeneration. Furthermore, repeated chemical treatment can induce structural changes in the protein and weaken the interfacial adhesion between keratin and the PLA/SBS matrix, resulting in a progressive reduction of available adsorption sites.

Despite this decline, the composites still retain measurable adsorption capacity after three regeneration cycles, demonstrating partial reusability.

Among the tested materials, PLA/SBS/K 10 offers a better balance between structural stability, thermal behavior, mechanical integrity, and reusability after regeneration cycles.

Future studies will be dedicated to optimizing keratin–polymer compatibility, kinetic, absorption isotherms as well as using advanced methods to elucidate the mechanism of Cr removal.

## 4. Conclusions

Keratin recovered from wool waste streams was investigated as a potential adsorbent material by valorizing it into PLA/SBS functional composites for the removal of Cr(VI) and total Cr from synthetic water.

The incorporation of keratin into PLA/SBS blends increased the roughness of surfaces and mechanical properties of the resulting materials. Keratin and PLA/SBS/Keratin samples exhibited moderate adsorption capacities. In the case of Cr(VI), the adsorption was particularly favored at acidic pH values, where the keratin surface becomes positively charged and can attract negatively charged chromium species. The functional groups in keratin (−SH, −COOH, −NH_2_) can bind Cr pollutant through electrostatic attraction, chelation, and hydrogen bonding. Thus, keratin introduces chemically active adsorption sites, while the PLA/SBS matrix provides mechanical integrity and thermal stability.

The thermal degradation analysis indicated that the PLA and SBS at different ratios and keratin loadings influenced the thermal stability and residue formation of the materials.

In addition, the incorporation of keratin into PLA/SBS 75/25 formulation demonstrated enhanced mechanical performance compared to neat blends, with increased storage modulus and stiffness, confirming the reinforcing effect of the keratin as a filler. The PLA/SBS/K composites showed potential for the removal of Cr from wastewater. Instead of being discarded or incinerated, keratin can contribute to waste minimization and supports circular economy principles through the conversion of low-cost waste into adsorbent materials.

## Figures and Tables

**Table 1:** Composition of the prepared formulations.

Polymeric System	PLA (wt%)	SBS (wt%)	K (wt%)
PLA/SBS 0/100	0	100	-
PLA/SBS 25/75	25	75	-
PLA/SBS 50/50	50	50	-
PLA/SBS 75/25	75	25	-
PLA/SBS/K 10	75	25	10
PLA/SBS/K 20	75	25	20
PLA/SBS/K 30	75	25	30
PLA/SBS 100/0	100	-	-

**Table 2:** Chemical composition of PLA/SBS/K 20 and PLA/SBS/K 30 composites.

Element	PLA/SBS/K 20			PLA/SBS/K 30		
	Weight (%)	Atomic (%)	Error (%)	Weight (%)	Atomic (%)	Error (%)
C K	59.9	66.9	8.9	54.8	63.6	10.0
O K	38.4	32.2	10.5	36.5	31.8	10.8
Na K	1.3	0.7	10.7	5.3	3.2	8.8
S K	0.4	0.2	19.1	3.0	1.3	6.0
Ca K	-	-	-	0.4	0.1	36.9

**Table 3:** Degradation temperature, weight loss, and residue for neat PLA, SBS, PLA/SBS blends, and PLA/SBS/K composites.

Sample	RT–170 °C		170–270 °C		270–370 °C		370–545 °C		545–700 °C	Residue at 700 °C	
	Weight Loss	T_max1_	Weight Loss	T_max2_	Weight Loss	T_max3_	Weight Loss	T_max4_	Weight Loss	N_2_	Air
	%	°C	%	°C	°C	°C	%	°C	%	%	%
PLA/SBS 100/0	6.44	122	0.24	-	91.33	353	1.23	-	0.25	0.51	0.03
PLA/SBS 75/25	5.99	120	1.22	-	67.43	341	24.70	448	0.25	0.41	0.02
PLA/SBS 50/50	1.92	87	2.35	-	44.11	335	51.03	448	0.27	0.32	0.03
PLA/SBS 25/75	1.72	103	2.80	-	35.13	326	59.84	448	0.18	0.33	0.02
PLA/SBS 0/100	0.07	0	0.25	-	4.00	0	95.36	444	0.21	0.11	0.03
PLA/SBS/K 10	6.93	109	44.66	261	14.25	285	20.22	441	1.56	12.38	8.15
PLA/SBS/K 20	8.08	126	28.01	243	21.59	290	18.91	443	2.77	20.64	15.34
PLA/SBS/K 30	8.92	118	25.78	243	22.57	291	20.49	444	2.47	19.77	15.08

**Table 4:** Kinetic parameters of Cr(VI) adsorption in the presence of keratin powder.

Adsorbate Concentration, mg L^−1^	q_e_, mg g^−1^	Pseudo-First Order			Pseudo-Second Order		
		q_max_, mg g^−1^	k_1_, min^−1^	R^2^	q_max_, mg g^−1^	k_2_, g mg^−1^ min^−1^	R^2^
10	3.304	3.774	0.015	0.808	4.575	0.576	0.820
20	4.004	4.659	0.038	0.899	7.471	0.177	0.975
40	15.203	15.707	0.028	0.990	23.068	0.004	0.992
70	26.801	24.164	0.046	0.968	30.312	0.0004	0.992
100	52.015	52.220	0.062	0.999	57.570	0.00004	0.999

**Table 5:** Comparison of the adsorption capacities of different adsorbents for removal of Cr(VI).

Material	q_max_, mg g^−1^	Reference
CS/clay composite bead	45.46	[77]
Coffee husk fiber/reinforced magnetic CS polyvinyl alcohol blend composite	14.22	[11]
CS/coated coconut shell composite	66.66	[6]
CS/MnO_2_/perlite composite	281.74	[33]
Cellulose composite aerogel adsorbent	411.12	[13]
Keratin extract	57.57	This study

**Table 6:** Chemical composition (wt%) of PLA/SBS/K 20 and PLA/SBS/K 30 composites after the total Cr adsorption and desorption.

Element	PLA/SBS/K 20		PLA/SBS/K 30	
	Adsorption	Desorption	Adsorption	Desorption
C K	65.6	63.1	57.4	57.7
O K	31.6	36.0	40.5	41.4
Na K	0.7	-	0.5	0.9
S K	1.6	0.9	0.9	-
Ca K	-	-	-	-
Si K	-	-	0.4	-
Cr K	0.4	-	0.3	-

## Data Availability

The original contributions presented in this study are included in the article. Further inquiries can be directed to the corresponding authors.
